# Development of a Reverse Transcription Recombinase Polymerase Amplification CRISPR/Cas12a Assay for Visual and Highly Specific Identification of Zika Virus

**DOI:** 10.1002/jmv.70917

**Published:** 2026-04-15

**Authors:** Xiao Cong, Xin Zhang, Feng Gu, Ningxin Tan, Shen Huang, Penghui Jia, Juan Su, Changyun Sun, Qiqi Tan, Ling Fang, Jieling Wang, Jin Yan, Chao Yu, Baisheng Li, Junqi Huang

**Affiliations:** ^1^ Organ Transplant Center, The First Affiliated Hospital Sun Yat‐Sen University Guangzhou Guangdong China; ^2^ Institute of Pathogenic Microbiology Guangdong Provincial Center for Disease Control and Prevention Guangzhou Guangdong China; ^3^ Guangdong Provincial Center for Disease Control and Prevention, Guangdong Provincial Key Laboratory of Pathogen Detection for Emerging Infectious Disease Response, Guangdong Workstation for Emerging Infectious Disease Control and Prevention Chinese Academy of Medical Sciences Guangzhou Guangdong China

**Keywords:** CRISPR/Cas12a, molecular diagnostics, point‐of‐care testing (POCT), recombinase polymerase amplification (RPA), Zika virus (ZIKV)

## Abstract

Zika virus (ZIKV), a single‐stranded positive‐sense RNA virus of the Flaviviridae family (*Flavivirus* genus), causes acute febrile illness and severe congenital anomalies. Serological cross‐reactivity with Dengue virus (DENV) and Chikungunya virus (CHIKV) complicates diagnosis, underscoring the urgency of developing specific point‐of‐care tests for early detection, outbreak mitigation, and reduced misdiagnosis risks. This study established and optimized an RPA–CRISPR/Cas12a assay for the rapid, visual, and highly specific detection of ZIKV. Primers and crRNAs targeting the highly conserved capsid (C) gene were designed, and the assay was systematically evaluated for its specificity, sensitivity, reproducibility, and clinical applicability. The RPA–CRISPR/Cas12a assay enabled naked‐eye detection under UV light within 35 min. It demonstrated single‐copy sensitivity (1 copy/μL), no cross‐reactivity with DENV 1–4, CHIKV, or Japanese encephalitis virus (JEV), and 100% concordance with RT‐qPCR in clinical validation. Repeatability tests showed low variability (coefficient of variation (C.V.) < 15%), confirming robust reproducibility. This instrument‐free platform integrates rapid visual detection, single‐copy sensitivity, high specificity, and field‐deployable features, making it particularly suitable for point‐of‐care testing (POCT) in resource‐limited settings. The developed assay provides critical support for early outbreak containment and prenatal screening in ZIKV‐endemic regions.

**Abbreviations**
CHIKV: Chikungunya virus
CR: concordance rate
CRISPR/Cas12a: clustered regularly interspaced short palindromic repeats/CRISPR‐associated protein 12a
C.V.: coefficient of variation
DENV: Dengue virus
dsDNA: double‐stranded DNA
JEV: Japanese encephalitis virus
LOD: limit of detection
ORF: open reading frame
PAM: protospacer adjacent motif
PHEIC: Public Health Emergency of International Concern
POCT: point‐of‐care testing
RNP: ribonucleoprotein
RPA: recombinase polymerase amplification
SSB: single‐stranded binding protein
ssDNA: single‐stranded DNA
ZIKV: Zika virus

## 1 Introduction

Zika virus (ZIKV), a single‐stranded positive‐sense RNA virus of the Flaviviridae family and *Flavivirus* genus [1], is an important mosquito‐borne pathogen associated with both acute febrile illness and severe congenital and neurological complications. Acute infection typically presents with mild and self‐limiting symptoms, including fever, rash, arthralgia, conjunctivitis, and headache [2, 3]. However, ZIKV infection during pregnancy can lead to serious fetal outcomes such as congenital microcephaly, ventriculomegaly, optic neuropathy, and other developmental abnormalities [4]. Since the World Health Organization declared the ZIKV outbreak a Public Health Emergency of International Concern (PHEIC) in 2016, the virus has continued to circulate at low levels in tropical and subtropical regions, posing an ongoing public health threat [5].

Accurate diagnosis of ZIKV infection remains challenging, particularly in regions where multiple arboviruses co‐circulate. Up to 80% of infections are asymptomatic, and early clinical manifestations overlap substantially with those of dengue and other flaviviral diseases. Moreover, serological assays frequently suffer from cross‐reactivity with Dengue virus (DENV) and Chikungunya virus (CHIKV), which complicates differential diagnosis and increases the risk of misclassification [6, 7, 8, 9, 10]. Although RT‐qPCR is considered the laboratory gold standard for ZIKV detection, its implementation depends on specialized instrumentation, trained personnel, and controlled laboratory conditions, which together limit its utility in resource‐limited and field settings [11]. These constraints highlight a critical need for rapid, sensitive, and field‐deployable molecular diagnostic methods for ZIKV.

Recent advances in isothermal amplification and CRISPR‐based nucleic acid detection have created new opportunities for decentralized molecular diagnostics. Recombinase polymerase amplification (RPA) enables rapid amplification of low‐copy nucleic acid targets at constant temperature without thermal cycling [12, 13]. CRISPR/Cas detection systems, particularly Cas12a, provide highly specific target recognition coupled with collateral trans‐cleavage activity for sensitive signal generation [14, 15, 16, 17, 18]. The integration of RPA with CRISPR/Cas12a therefore represents a promising strategy for rapid and portable pathogen detection. However, despite growing interest in CRISPR‐based diagnostics, few studies have systematically optimized an integrated RPA–CRISPR/Cas12a assay specifically for ZIKV, and comprehensive validation against closely related arboviruses and clinical samples remains limited.

Target selection is another critical determinant of assay specificity. Compared with the envelope (E) and non‐structural protein 1 (NS1) regions, the capsid (C) gene has been reported to show higher specificity for distinguishing ZIKV from other flaviviruses and exhibits strong evolutionary conservation, making it a suitable target for diagnostic assay development [19].

In this study, we developed and systematically evaluated an RPA–CRISPR/Cas12a detection platform targeting the conserved ZIKV C gene. The assay couples one‐step RT‐RPA amplification with Cas12a‐mediated trans‐cleavage for real‐time fluorescence readout and visual detection under UV illumination. We further assessed its analytical sensitivity, specificity against related arboviruses, repeatability, and clinical performance. This platform provides a rapid, sensitive, and highly specific approach for ZIKV detection and supports decentralized testing and early outbreak response.

## 2 Materials and Methods

### 2.1 Major Instruments, Equipment, and Clinical Samples

The viral nucleic acid extraction reagents and instruments were purchased from Xi'an TIANLONG Technology Co. Ltd. The instrument used was the GeneRotex series fully automated nucleic acid extractor. RNA Rapid Isothermal Amplification Kit (Basic) was purchased from Guangzhou Amplification Future Co. Ltd. The LbCas12a Nuclease (Cat. Z03753) was purchased from Nanjing GenScript Biotech Co. Ltd. The fluorescence acquisition instrument used was the CFX series real‐time fluorescence quantitative PCR system, purchased from Bio‐Rad Laboratories (Shanghai, China) Co. Ltd. PCR Product Purification Kit was purchased from Beyotime Biotechnology (Shanghai, China). All clinical samples were provided by the Guangdong Provincial Center for Disease Control and Prevention.

### 2.2 Design of Primers and crRNAs

Before establishing the RPA amplification system, the conservation of the ZIKV C gene was assessed using SnapGene (Insightful Science, USA), and the highly conserved region was selected as the amplification target. Five primer pairs targeting the ZIKV C gene (GenBank: KU509998.3) were designed following standard RPA primer design principles as described in the manufacturer's guidelines [20], and their specificity was confirmed using NCBI BLAST. All primers were synthesized by Nanjing GenScript Biotech Co. Ltd.

For CRISPR/Cas12a detection, crRNAs corresponding to each RPA amplicon were designed according to the Cas12a protospacer adjacent motif (PAM) requirement and synthesized by Nanjing GenScript Biotech Co. Ltd. The full crRNA sequences used in this study are provided in Table 1. A fluorescent ssDNA reporter containing a FAM fluorophore and a BHQ1 quencher was also synthesized by Nanjing GenScript Biotech Co. Ltd.

**TABLE 1:** Primers, crRNAs, and fluorescent ssDNA reporter sequences used in RPA–CRISPR/Cas12a detection system.

Primers/crRNAs name	Sequence (5′−3′)	Location	Product size (bp)
ZIKV C 1 F	GTCTTGGCAATTCTAGCCTTTTTGAGATTC	139–168	165
ZIKV C 1 R	CTTCTCCTTCCTAGCATTGATTATTCTCAG	303–274	
LbCas12a crRNA ZC1	UAAUUUCUACUAAGUGUAGAUcccacugaaccccaucuauu		
ZIKV C 2 F	GTCTTGGCAATTCTAGCCTTTTTGAGATTCAC	139–170	173
ZIKV C 2 R	CGTCTCTTCTTCTCCTTCCTAGCATTGATTAT	311–280	
LbCas12a crRNA ZC2	UAAUUUCUACUAAGUGUAGAUCAUAGCCUCUUUUUUCCCCA		
ZIKV C 3 F	GGTCTTGGCAATTCTAGCCTTTTTGAGATTCAC	138–170	176
ZIKV C 3 R	CTCGTCTCTTCTTCTCCTTCCTAGCATTGATTA	313–281	
LbCas12a crRNA ZC3	UAAUUUCUACUAAGUGUAGAUUUAUUUCCAUAGCCUCUUUU		
ZIKV C 4 F	CTTGGCAATTCTAGCCTTTTTGAGATTCAC	141–170	170
ZIKV C 4 R	GTCTCTTCTTCTCCTTCCTAGCATTGATTA	310–281	
LbCas12a crRNA ZC4	UAAUUUCUACUAAGUGUAGAUUUGAACUUCUUUAUUAUUUC		
ZIKV C 5 F	GAGGATTCCGGATTGTCAATATGCTAAAAC	26–55	145
ZIKV C 5 R	GTGAATCTCAAAAAGGCTAGAATTGCCAAGAC	170–139	
LbCas12a crRNA ZC5	UAAUUUCUACUAAGUGUAGAUGGGGCUUGAAGAGGCUGCCA		
Fluorescent ssDNA reporter	FAM‐TTATT‐BHQ1		

### 2.3 Establishment of the Recombinase Polymerase Amplification System

This assay employed the RNA Rapid Isothermal Amplification Kit (Basic) (Amplification Future, China), which contains both reverse transcriptase and DNA polymerase. Therefore, viral RNA served directly as the template, and the reverse transcription and amplification steps were carried out simultaneously in a one‐step RT‐RPA reaction without separate cDNA synthesis. The reaction was performed in a total volume of 50 µL. After opening the tube containing the freeze‐dried reagents, 29.5 µL of Rehydration buffer was added and mixed thoroughly. Subsequently, 2 µL each of the RPA forward primer (Primer‐F) and RPA reverse primer (Primer‐R) (10 µM), 14.1 µL of RNA template and ddH₂O, and 2.5 µL of MgOAc (280 mM) were added. The Primer‐F and Primer‐R sequences correspond to the ZIKV C gene primers listed in Table 1. The reaction was immediately incubated at 37°C for 20 min in a dry‐bath heating block. Nuclease‐free water served as the no‐template control (NTC) in all RT‐RPA reactions.

### 2.4 Purification, Sequencing, and Validation of RPA Amplicons

RPA products were purified using a PCR Product Purification Kit (Beyotime, China) following the manufacturer's instructions, and subsequently verified by agarose gel electrophoresis. Although CRISPR–Cas12a assays can be performed using unpurified RPA products, purification was included to eliminate potential inhibitors and to facilitate a controlled assessment of whether RPA reaction components affect downstream Cas12a‐based detection. The purified DNA was used directly for subsequent CRISPR/Cas12a analysis.

To validate the specificity of the RPA amplification products, amplicons generated by each primer set were subjected to Sanger sequencing. The purified RPA products were sent to GenScript (Nanjing, China) for sequencing using the corresponding amplification primers. The obtained sequencing reads were assembled and aligned to the reference ZIKV template sequence (GenBank accession no. KU509998.3) that was used for primer design. Sequence alignment was performed using SnapGene software.

### 2.5 Development of an RPA–CRISPR/Cas12a Detection System

An RPA–CRISPR/Cas12a–based detection system was developed for ZIKV by coupling recombinase polymerase amplification with Cas12a‐mediated trans‐cleavage activity. Briefly, the CRISPR/Cas12a reaction was performed using LbCas12a nuclease, corresponding crRNA, and a FAM‐labeled single‐stranded DNA reporter in Cas12a reaction buffer. The Cas12a–crRNA ribonucleoprotein complex was preassembled prior to the detection reaction. Fluorescence signals generated by trans‐cleavage of the reporter were monitored under isothermal conditions using a CXF instrument, with fluorescence acquisition at regular intervals in the FAM channel.

Nuclease‐free water was used as the negative control (NTC) throughout all CRISPR/Cas12a detection assays. To ensure optimal assay performance, the working concentrations of LbCas12a nuclease, crRNA, and the ssDNA reporter were determined based on the manufacturer's recommendations and previously reported RPA–CRISPR/Cas12a systems [20, 21, 22, 23].

### 2.6 Construction of a Positive Control Plasmid

We commissioned GenScript (Nanjing, China) to synthesize the full‐length gene of the ZIKV C protein (GenBank: KU509998.3) and clone it into the pUC57 vector to construct a standard positive control plasmid for validating the RPA–CRISPR/Cas12a system. The lyophilized plasmid DNA (4 µg) was reconstituted in nuclease‐free water and serially diluted to obtain working concentrations of 10⁵, 10⁴, 10³, 10², 10¹, and 10° copies/µL based on the calculated plasmid copy number. The 0.1 copies/µL dilution was prepared by an additional 10‐fold dilution of the 1 copy/µL standard, representing a theoretical copy number commonly used in analytical sensitivity evaluation.

### 2.7 The Specificity Analysis of the RPA–CRISPR/Cas12a Detection System

Specificity analysis is a crucial step in evaluating the performance of a detection method. To assess the specificity of the RPA–CRISPR/Cas12a detection system established in this study, we tested seven viruses stored in our laboratory, including Dengue virus‐1 (DENV‐1), Dengue virus‐2 (DENV‐2), Dengue virus‐3 (DENV‐3), Dengue virus‐4 (DENV‐4), Chikungunya virus (CHIKV), Japanese encephalitis virus (JEV), and Zika virus (ZIKV), along with a negative control. The method is considered to have good specificity only if ZIKV is identified as positive. Nuclease‐free water was consistently used as the NTC in the CRISPR/Cas12a detection assays.

### 2.8 The Sensitivity Analysis of the RPA–CRISPR/Cas12a Detection System

Next, we evaluated the sensitivity of the RPA–CRISPR/Cas12a detection system developed in this study. Standard positive plasmids at concentrations of 10^5^, 10^4^, 10^3^, 10^2^, 10^1^, 10°, and 10^−1^ copies/µL were tested, along with a negative control group, to determine the minimum detectable concentration of the assay. Nuclease‐free water was consistently used as the NTC in the CRISPR/Cas12a detection assays.

### 2.9 Repeatability Analysis of the RPA–CRISPR/Cas12a Detection System

To evaluate the repeatability of the RPA–CRISPR/Cas12a detection system, a series of standard positive plasmid concentrations ranging from 10⁵ to 10⁻¹ copies/µL was tested. Each concentration was analyzed in triplicate under identical experimental conditions, and a negative control was included. The coefficient of variation (C.V.), calculated as the standard deviation divided by the mean, was used to assess the repeatability of the assay across different target concentrations. Nuclease‐free water was consistently used as the NTC in the CRISPR/Cas12a detection assays.

### 2.10 Clinical Sample Detection and Analysis

To evaluate the clinical applicability of the detection method established in this study, we tested 39 serum clinical samples stored at the Guangdong Provincial Center for Disease Control and Prevention (including both ZIKV‐positive and ZIKV‐negative samples) using both RT‐qPCR and RPA–CRISPR/Cas12a detection methods. The concordance rate (CR) between the results of the two methods was compared to assess the clinical application value of the RPA–CRISPR/Cas12a detection method. An additional no‐template control (NTC) was processed in parallel with clinical samples.

### 2.11 RT‐qPCR Detection of ZIKV and Other Viruses (Commercial Assay)

Clinical samples were tested using commercial real‐time RT‐PCR detection kits specific for ZIKV and other arboviruses (BioGerm, China), following the manufacturer's instructions. All assays are designed to target conserved regions of the respective viral genomes; however, the primer and probe sequences are proprietary and therefore not publicly disclosed by the manufacturer. All RT‐qPCR reactions were performed on a Bio‐Rad CFX real‐time PCR system under the reaction conditions specified in the corresponding kits. Cycle threshold (Ct) values were interpreted according to the manufacturer's criteria, with Ct values < 38 considered positive and Ct values ≥ 38 considered negative. Positive and negative controls provided with each kit were included in every run to ensure assay validity and reliability. The results obtained from these commercial RT‐qPCR assays were used as the reference standard for evaluating and comparing the performance of the RPA–CRISPR/Cas12a detection system.

### 2.12 Statistical Analysis

All statistical analyses were performed using GraphPad Prism 8. Error bars represent mean ± SD, *p*‐values are displayed as ns for *p* > 0.05, *p < 0.05, **p < 0.01, ***p < 0.001, and ****p < 0.0001.

## 3 Results

### 3.1 Primer Screening

An overview of the RPA–CRISPR/Cas12a detection workflow is provided in Supporting Figure S1, which illustrates how RPA amplifies the target sequence under isothermal conditions and how the resulting amplicons activate Cas12a through crRNA‐guided recognition, leading to trans‐cleavage of fluorescent ssDNA reporter and generation of detectable fluorescence signals.

**FIGURE 1:**
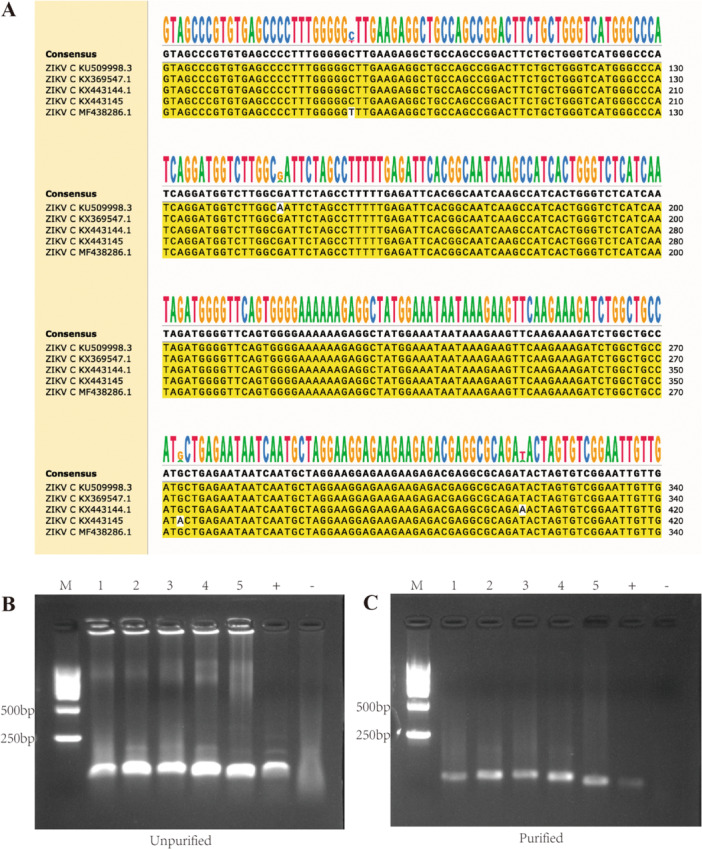
Establishment of the RPA system. (A) Multiple sequence alignment of ZIKV C gene sequences from different isolates. (B) Nucleic acid gel electrophoresis of unpurified RPA products. (C) Nucleic acid gel electrophoresis of purified RPA products. Lane M, marker; lane 1–5, amplification products of RPA primer pairs 1–5; lane +, positive control; lane −, negative control.

To identify suitable amplification targets, five ZIKV C gene sequences (accession numbers KU509998.3, KX369547.1, KX443144.1, KX443145, and MF438286.1) were retrieved from NCBI and aligned using SnapGene (Insightful Science, USA). As shown in Figure 1A, the C gene exhibited high sequence conservation across strains, with only minimal nucleotide variation, confirming its suitability as a diagnostic target for ZIKV. Next, we used the ZIKV strain stored in our laboratory as a template to evaluate the performance of the designed RPA primers targeting the C gene. To compare the effects of purified vs. unpurified RPA amplification products in subsequent CRISPR/Cas12a detection, both products were subjected to agarose gel electrophoresis. As shown in Figure 1B (unpurified) and Figure 1C (purified), all five primer sets successfully amplified the target sequences, although the purified products exhibited lower concentration than the unpurified ones. To further verify the specificity and correctness of the amplification, the amplicons were subjected to sequencing analysis. Sequence alignment demonstrated that the amplified sequences were identical to the template sequences used for primer design (Supporting Figure S2), confirming that the observed amplification products originated from the intended target rather than from nonspecific amplification.

### 3.2 Construction and Optimization of the RPA–CRISPR/Cas12a Detection System

We subjected both unpurified and purified RPA products to CRISPR/Cas12a detection. The real‐time fluorescence results (Supporting Figure S3) showed that all five primer–crRNA combinations produced detectable signals, and the fluorescence intensities of unpurified RPA products were consistently higher than those of purified products. Although no significant difference was observed in the endpoint fluorescence values between purified and unpurified products, the unpurified RPA products consistently exhibited higher fluorescence signals, indicating that unpurified RPA products are more suitable for direct CRISPR/Cas12a detection without the need for additional purification steps. Among the five primer–crRNA combinations, pair No. 2 showed the strongest fluorescence and was therefore selected for subsequent assay optimization. As shown in the fluorescence kinetics (Supporting Figure S3A), the fluorescence intensity exceeded 10 000 RFU by cycle 30. Therefore, the CRISPR/Cas12a reaction time was optimized by setting the detection duration to 30 cycles, with each cycle lasting 30 s (total 15 min).

### 3.3 Specificity Analysis of the RPA–CRISPR/Cas12a Detection System

**FIGURE 2:**
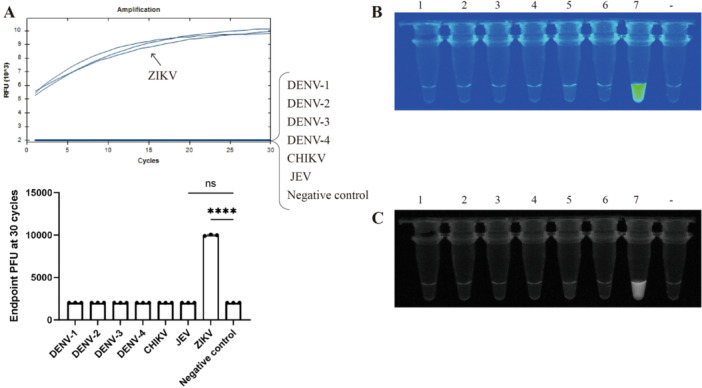
Fluorescence detection results of RPA–CRISPR/Cas12a for seven different arboviruses. (A) Fluorescence acquisition results of RPA–CRISPR/Cas12a detection for seven different arboviruses (****p < 0.0001; ns, not significant). (B and C) Fluorescence results of RPA–CRISPR/Cas12a detection for seven different arboviruses under UV light. Lane 1, DENV‐1; lane 2, DENV‐2; lane 3: DENV‐3; lane 4: DENV‐4; lane 5: CHIKV; lane 6: JEV; lane 7: ZIKV; lane −, negative control.

Good specificity is a fundamental requirement for establishing a pathogen detection method. After constructing the RPA–CRISPR/Cas12a detection system, we validated its specificity using seven different arboviruses: Dengue virus‐1 (DENV‐1), Dengue virus‐2 (DENV‐2), Dengue virus‐3 (DENV‐3), Dengue virus‐4 (DENV‐4), Chikungunya virus (CHIKV), Japanese encephalitis virus (JEV), and Zika virus (ZIKV). All seven viruses were provided by the Guangdong Provincial Center for Disease Control and Prevention. The detection results, as shown in Figure 2A, demonstrated that only ZIKV exhibited a significant fluorescence curve. Under UV light, fluorescence was observed only in the ZIKV reaction tube (Figure 2B,C). For the other six viruses, we also performed validation using RT‐qPCR, with the detection results presented in Supporting Figure S4. Therefore, we conclude that this method has excellent specificity and does not cross‐react with other arboviruses.

### 3.4 Sensitivity Analysis of the RPA–CRISPR/Cas12a Detection System

**FIGURE 3:**
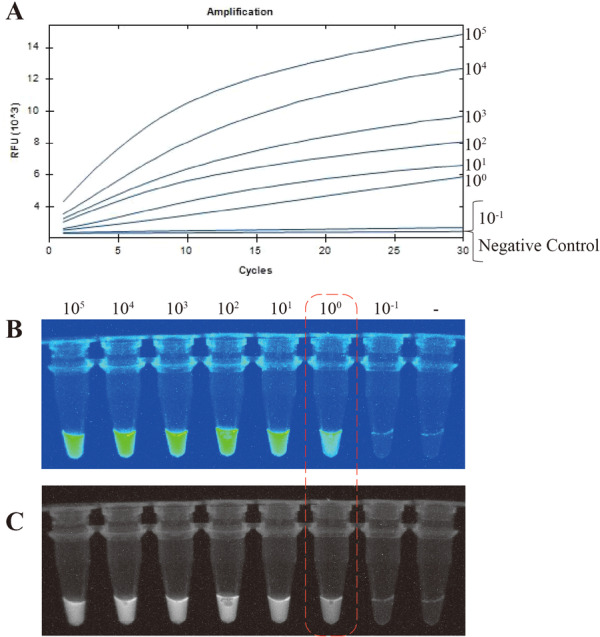
Fluorescence detection results of RPA–CRISPR/Cas12a for seven positive plasmid samples at different concentrations.(A) Fluorescence acquisition results of RPA–CRISPR/Cas12a detection for seven positive plasmid samples at different concentrations. (B and C) Fluorescence results of RPA–CRISPR/Cas12a detection for seven positive plasmid samples at different concentrations under UV light. Lane −, negative control.

Sensitivity is an indicator that evaluates the ability of a detection method to distinguish between different concentrations of viruses with precision. In this study, we prepared serially diluted standard plasmid samples at concentrations of 10⁵, 10⁴, 10³, 10², 10¹, 10⁰, and 10⁻¹ copies/μL, along with a negative control group, to determine the limit of detection (LOD) of the developed RPA–CRISPR/Cas12a assay. As shown in Figure 3A, our RPA–CRISPR/Cas12a system consistently detected significant fluorescence signals even at 10¹ copies/µL. However, no fluorescence was observed for the 10⁻¹ copies/µL sample, mirroring the negative control, thereby validating the assay's specificity. Moreover, consistent results were obtained between real‐time fluorescence and visual detection modalities in the ZIKV RPA–CRISPR/Cas12a system, where fluorescence was observable at 10° copies/µL but absent in the 10^−1^ copies/µL reaction tube (Figure 3B,C). These findings establish the LOD of our method as 10° copies/µL (equivalent to 1 copy/µL). Meanwhile, we found that in previous studies, the LOD of traditional RT‐qPCR for ZIKV was confirmed to be 10¹ copies/µL [5], which indicates that the detection method we established is more sensitive than traditional RT‐qPCR.

### 3.5 Repeatability Analysis of the RPA–CRISPR/Cas12a Detection System

To comprehensively evaluate the repeatability of the RPA–CRISPR/Cas12a detection system, serial dilutions of standard positive plasmids ranging from 10⁵ to 10^−1^ copies/µL were tested in triplicate, together with a no‐template control (NTC). All reactions were performed under identical conditions, and fluorescence signals were recorded in real time. As shown in Supporting Figure S5, consistent amplification curves were observed across all tested concentrations, with minimal variation among the three independent replicates at each concentration. Quantitative analysis of endpoint fluorescence intensities demonstrated a concentration‐dependent decrease in signal strength. The coefficients of variation (C.V., calculated as SD/mean) for all concentrations were below 15%, indicating good repeatability across the entire dynamic range of the assay. At lower target concentrations (10¹ and 10⁰ copies/µL), slightly increased variability was observed, which is expected for reactions approaching the detection limit; however, the overall fluorescence trends remained highly consistent among replicates. In contrast, the NTC group consistently showed low and stable background fluorescence without significant fluctuation. Collectively, these results demonstrate that the RPA–CRISPR/Cas12a detection system exhibits robust and reproducible performance across a wide range of target concentrations, supporting the reliability of the assay for quantitative and qualitative detection.

### 3.6 Clinical Application of RPA–CRISPR/Cas12a

We evaluated a total of 39 clinical serum samples stored at the Pathogen Microbiology Laboratory of the Guangdong Provincial Center for Disease Control and Prevention. These samples were collected from imported cases in Guangdong Province and included both ZIKV‐positive and ZIKV‐negative specimens. All samples were tested in parallel using both RT‐qPCR and the RPA–CRISPR/Cas12a detection method, with a negative control included in each run.

Table 2 presents the comparative analysis of detection results obtained by the two methods for all 39 clinical samples. Complete concordance (100%) was observed between RT‐qPCR and RPA–CRISPR/Cas12a, with no false‐positive or false‐negative results. The final dataset comprised five positive samples, 34 negative samples, and a negative control. Detailed identification information for the five positive samples is provided in Table 3.

**TABLE 2:** Comparison between the RPA–CRISPR/Cas12a and RT‐qPCR for detection of ZIKV.

		RT‐qPCR			CR (%)
		+	−	Total	
RPA–CRISPR/Cas12a	+	5	0	5	100
	−	0	34	34	
	Total	5	34	39	

Abbreviations: CR, concordance rate; CR, (true positive + true negative)/total × 100%; +, positive sample; −, negative sample.

**TABLE 3:** Five positive clinical samples information.

Sample ID	Genotype	Sample type
Z16019	PRVABC59 (Asian genotype)	Serum
Z16461	PRVABC59 (Asian genotype)	Serum
D230073	PRVABC59 (Asian genotype)	Serum
D230074	PRVABC59 (Asian genotype)	Serum
D230394	PRVABC59 (Asian genotype)	Serum

**FIGURE 4:**
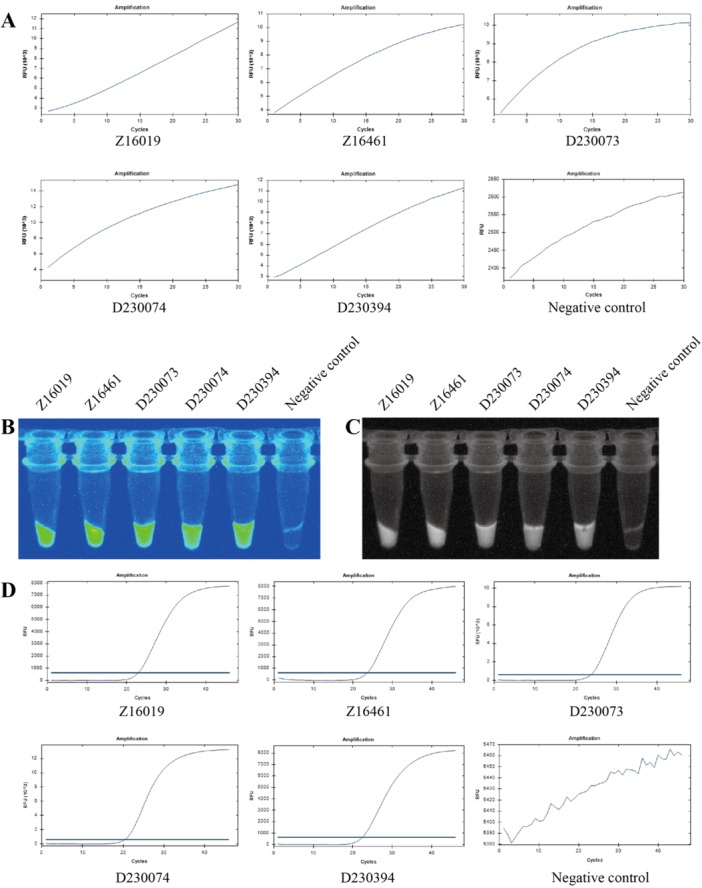
Comparison of detection results between RPA–CRISPR/Cas12a and RT‐qPCR for five positive clinical samples and a negative control. (A) Fluorescence acquisition results of RPA–CRISPR/Cas12a for the five positive clinical samples and a negative control. (B and C) Visual detection results under ultraviolet light for the five positive clinical samples and a negative control using RPA–CRISPR/Cas12a. (D) Detection results of RT‐qPCR for the five positive clinical samples and a negative control.

Figure 4A–C display the RPA–CRISPR/Cas12a detection results for the five ZIKV‐positive clinical samples and a negative control, while the corresponding RT‐qPCR amplification results are shown in Figure 4D. To fully present the dataset and address the negative samples, real‐time fluorescence amplification curves for the remaining 34 ZIKV‐negative clinical samples are provided in Supporting Figure S6. All negative samples consistently exhibited low and stable background fluorescence signals without detectable amplification, further confirming the reliability and clinical applicability of the RPA–CRISPR/Cas12a assay.

Together, these results demonstrate that the established RPA–CRISPR/Cas12a detection method shows excellent agreement with RT‐qPCR and possesses significant potential for clinical application.

## 4 Discussion

In this study, we established a rapid, sensitive, and highly specific RPA–CRISPR/Cas12a assay targeting the conserved ZIKV C gene. The assay achieved single‐copy sensitivity, exhibited no cross‐reactivity with DENV1–4, CHIKV, or JEV, and showed complete concordance with RT‐qPCR in clinical samples, demonstrating strong potential for early ZIKV detection. Given that ZIKV can infect immune‐privileged tissues, persist in sites such as the testes and eyes, and cause severe complications, including congenital defects, early, and accessible diagnosis remains crucial for outbreak control and protection of high‐risk populations [24, 25]. Moreover, with up to 80% of ZIKV infections being asymptomatic and current diagnostic methods relying heavily on laboratory infrastructure, the development of decentralized, rapid molecular assays is essential for timely identification of cases, particularly in resource‐limited settings and during border surveillance [26].

In recent years, gene editing technologies, represented by CRISPR/Cas, have brought revolutionary advancements to biotechnology. CRISPR‐based nucleic acid detection technology, characterized by its speed, accuracy, sensitivity, and cost‐effectiveness, is driving a transformation in molecular diagnostics. It has already been successfully applied in various fields, including infectious diseases, genetic disorders, cancer gene mutation diagnosis, and food safety. Numerous studies have demonstrated the excellent specificity and sensitivity of CRISPR/Cas12a when combined with RPA [20, 21, 22, 23]. The setup only requires basic laboratory equipment, such as a constant‐temperature heater, micropipettes, and a portable UV source, which are inexpensive and widely available. Additionally, the total cost per test is approximately 4–6 USD, which is lower than that of conventional RT‐qPCR (10–15 USD) or droplet digital PCR (20–30 USD per test). These features highlight the economic and practical advantages of the RPA–CRISPR/Cas12a platform for large‐scale screening and point‐of‐care testing in resource‐limited settings.

Compared with previously reported ZIKV detection methods, our RPA–CRISPR/Cas12a assay offers a balanced combination of sensitivity, specificity, speed, and operational simplicity. Conventional RT‐qPCR and ddPCR remain highly sensitive (down to 10^−1^ copies) [5], but their reliance on thermal cyclers, specialized personnel, and centralized laboratories limits their applicability in resource‐constrained settings. Isothermal methods such as RT‐LAMP and RT‐RPA have reduced equipment requirements, yet their performance varies: RT‐LAMP typically needs higher temperatures (≈63°C) and longer reaction times, whereas earlier RT‐RPA assays reported lower sensitivity or lacked comprehensive specificity evaluation [27]. Serological assays, including IgM ELISA and NS1‐based formats, provide useful complementary information but suffer from delayed antibody kinetics and cross‐reactivity with related flaviviruses [28, 29]. In contrast, the RPA–CRISPR/Cas12a system developed in this study achieves single‐copy sensitivity, demonstrates excellent specificity without cross‐reactivity to DENV1–4, CHIKV, or JEV, and produces visually interpretable results within 35 min under isothermal conditions. Its minimal equipment requirement and rapid workflow make it particularly suitable for decentralized surveillance, field deployment, and early detection during outbreaks.

Overall, our findings demonstrate that the RT‐RPA–CRISPR/Cas12a assay constitutes a promising molecular diagnostic tool that combines speed, sensitivity, and operational simplicity. By enabling rapid and decentralized detection, it may support outbreak surveillance, early case identification, and public health responses, particularly in regions lacking advanced laboratory infrastructure.

## Author Contributions

**Xiao Cong** spearheaded the investigation, devised the methodology, and authored the initial draft. **Xin Zhang** performed the formal analysis and validated the findings. **Feng Gu** was primarily responsible for conducting experiments and collecting data. **Ningxin Tan** was primarily responsible for conducting experiments and collecting data. **Shen Huang** was primarily responsible for conducting experiments and collecting data. **Penghui Jia** not only facilitated the acquisition of essential viral resources but also engaged in the project's investigative efforts and oversight. **Juan Su** contributed to resource preparation and investigative tasks. **Changyun Sun** contributed to resource preparation and investigative tasks. **Qiqi Tan** contributed to resource preparation and investigative tasks. **Ling Fang** contributed to resource preparation and investigative tasks. **Jieling Wang** contributed to resource preparation and investigative tasks. **Jin Yan** contributed to resource preparation and investigative tasks. **Chao Yu** contributed to resource preparation and investigative tasks. **Baisheng Li** provided support in data collection. **Junqi Huang** took the helm in conceptualizing the study, acquiring funding, and overseeing project management. He also played a significant role in manuscript revision and editorial processes.

## Ethics Statement

The serum samples used in this study were sourced from anonymized sample archives stored by various regional Centers for Disease Control and Prevention (CDC) between 2016 and 2023 and were transferred to the Guangdong Provincial Center for Disease Control and Prevention for storage and research purposes. All samples were labeled with identification numbers, rendering them untraceable to individual identities. The research did not involve the analysis of sensitive information, and therefore, written informed consent was waived. This study solely conducted detection and analysis on desensitized samples and did not involve the collection of new human samples or the association of individual private data. The data collection and sample acquisition procedures complied with the Zika Virus Disease Prevention and Control Plan approved by the National Health and Family Planning Commission of the People's Republic of China. Consequently, this study posed minimal risk to patients and did not adversely affect their rights or health.

## Conflicts of Interest

The authors declare no conflicts of interest.

## Supporting information

Supporting File 1

Supporting File 2

Supporting File 3

Supporting File 4

Supporting File 5

Supporting File 6

Supporting File 7

Supporting File 8

## Data Availability

All data generated or analyzed during this study are included in this published article [and its Supporting Information Files].
