# Supplementary figures and images for: Development of a Reverse Transcription Recombinase Polymerase Amplification CRISPR/Cas12a Assay for Visual and Highly Specific Identification of Zika Virus
Source: J Med Virol. 2026 Apr 15;98(4):e70917. doi: 10.1002/jmv.70917 (PMC13080280; doi:10.1002/jmv.70917)

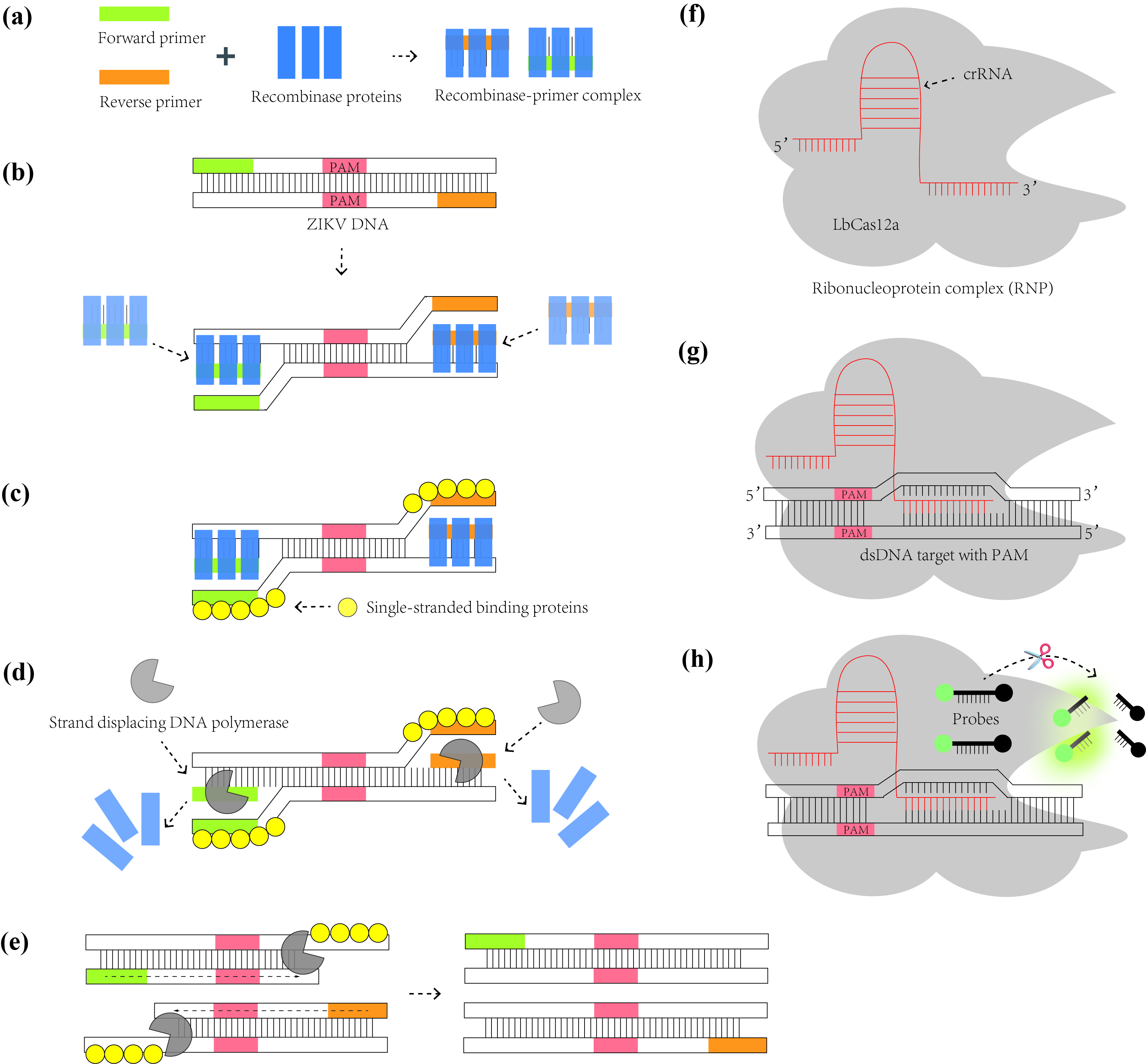

Supplement: Supplementary file 1 — Supporting File 1 [file JMV-98-e70917-s002.jpg]

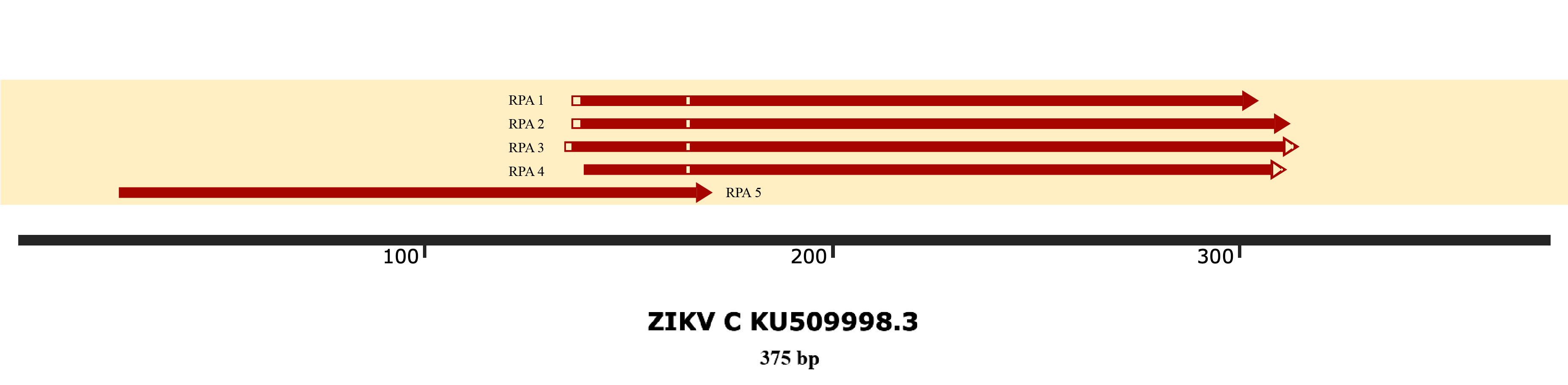

Supplement: Supplementary file 2 — Supporting File 2 [file JMV-98-e70917-s005.jpg]

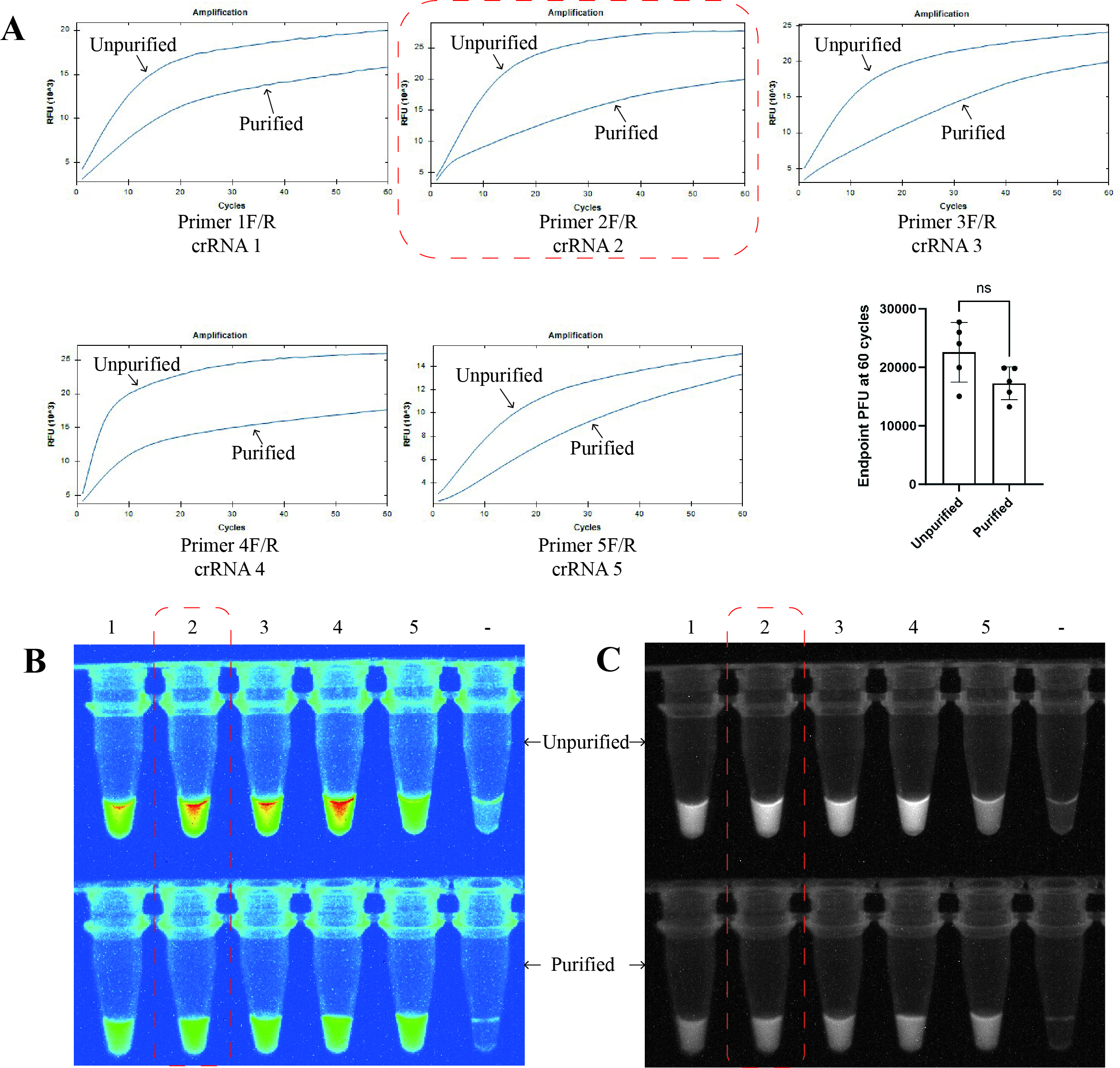

Supplement: Supplementary file 3 — Supporting File 3 [file JMV-98-e70917-s003.jpg]

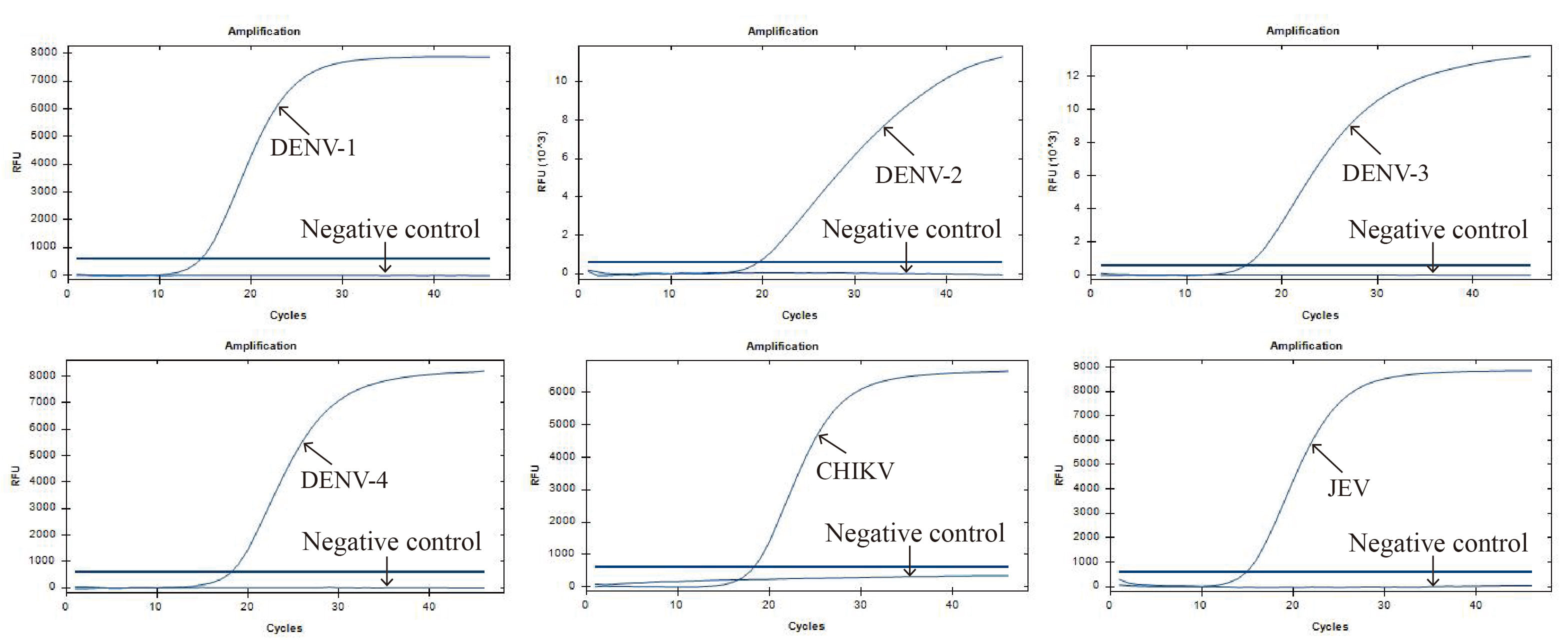

Supplement: Supplementary file 4 — Supporting File 4 [file JMV-98-e70917-s007.jpg]

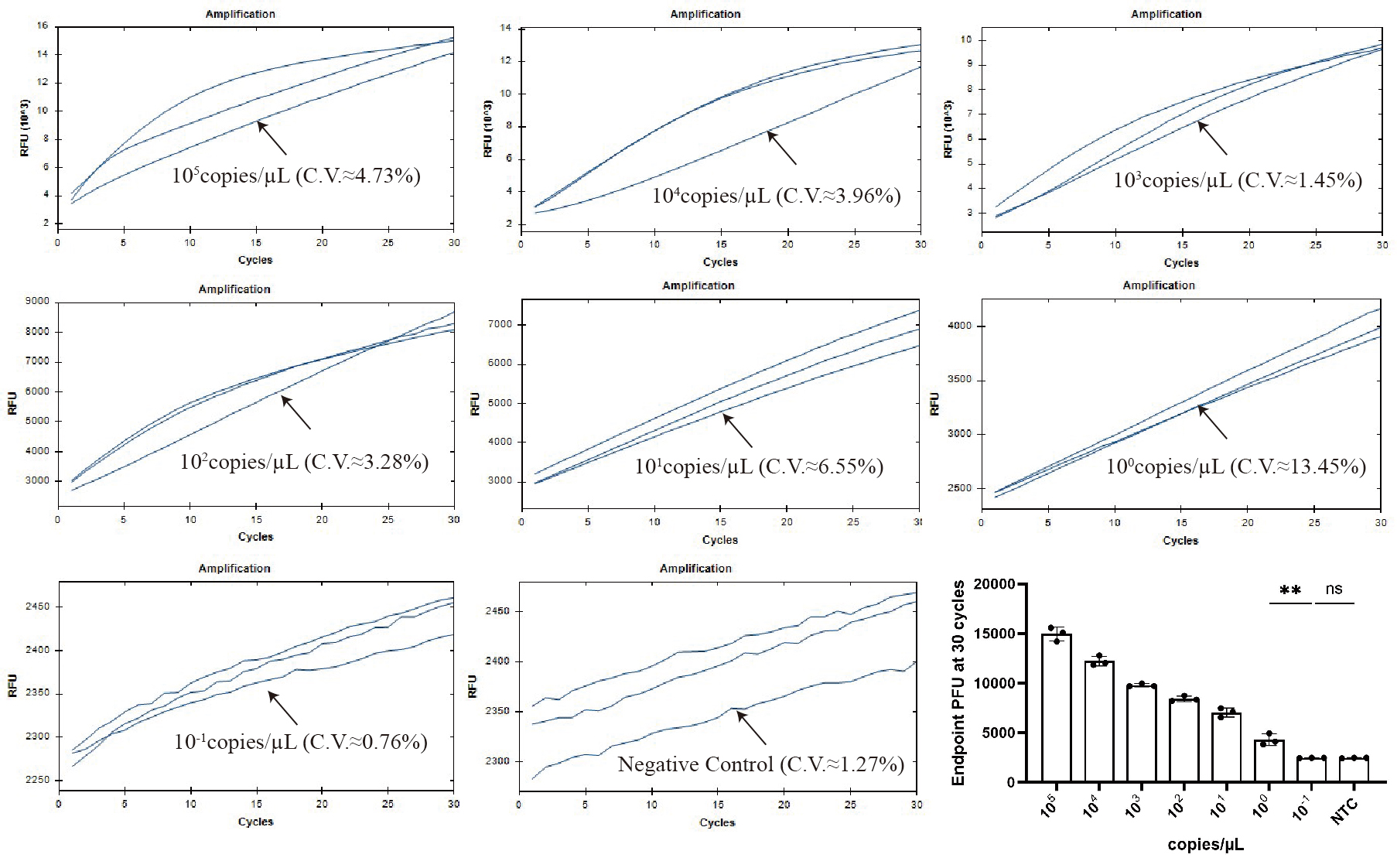

Supplement: Supplementary file 5 — Supporting File 5 [file JMV-98-e70917-s008.jpg]

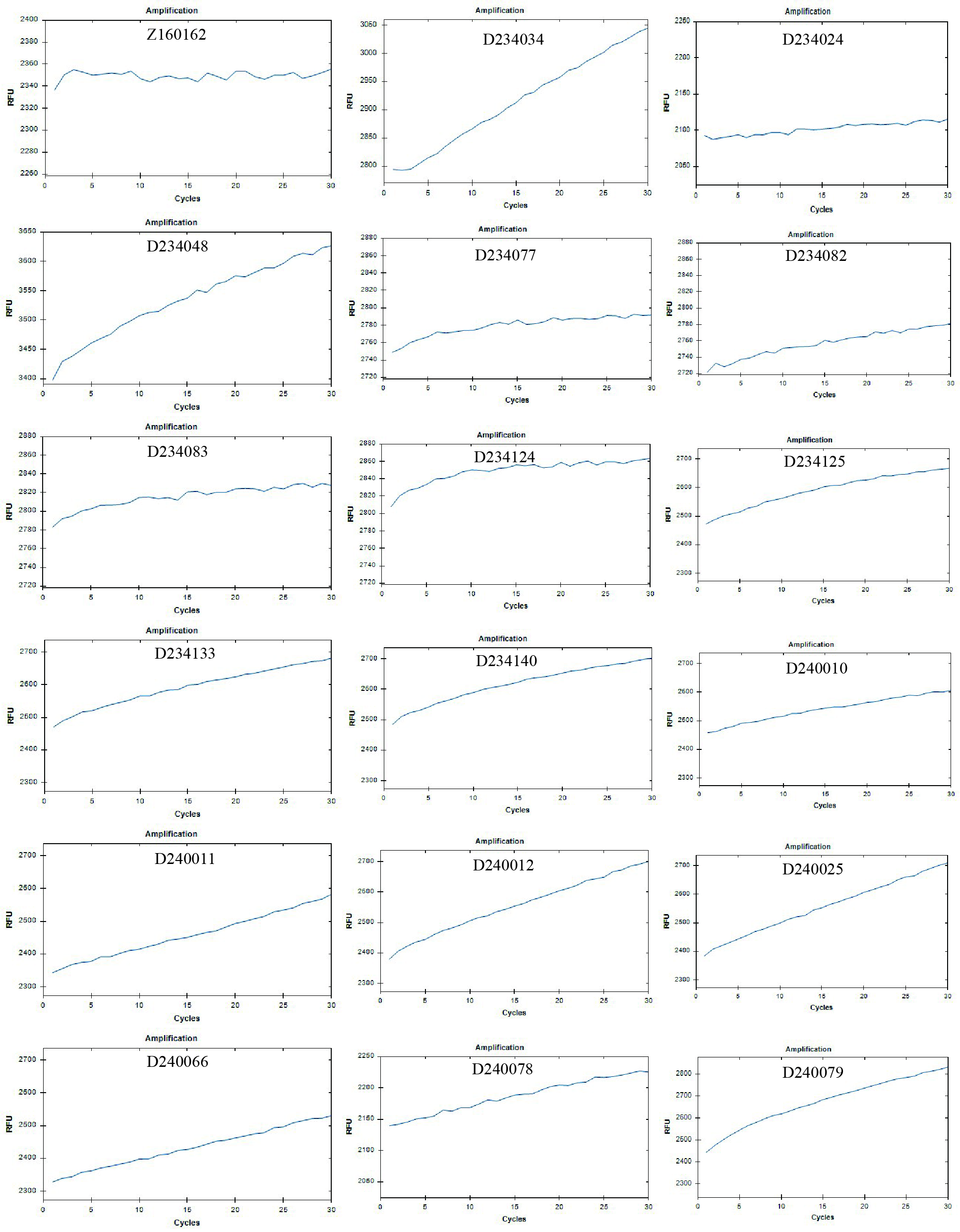

Supplement: Supplementary file 6 — Supporting File 6 [file JMV-98-e70917-s001.jpg]

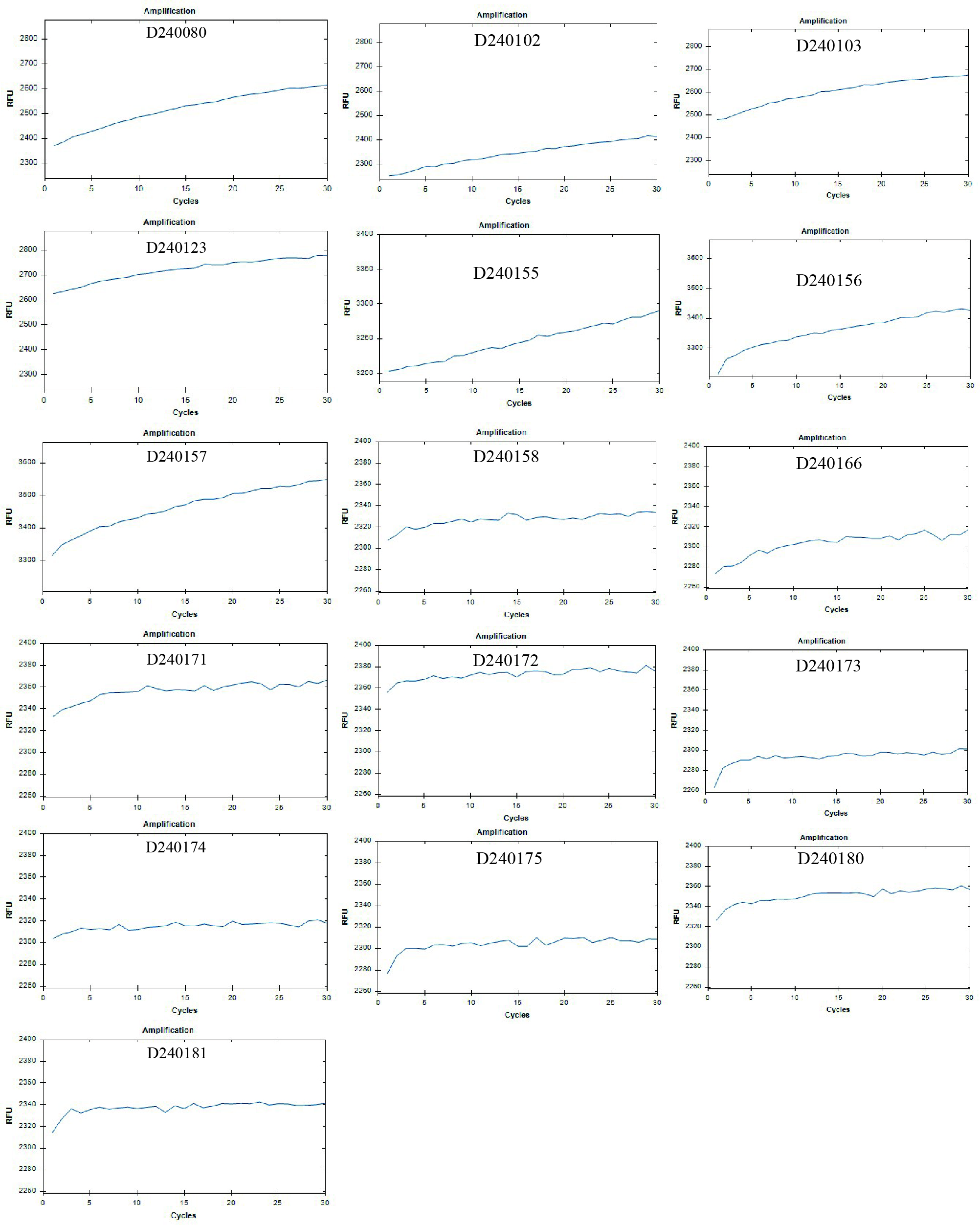

Supplement: Supplementary file 7 — Supporting File 7 [file JMV-98-e70917-s006.jpg]
