# Supplementary material for: Development of a Reverse Transcription Recombinase Polymerase Amplification CRISPR/Cas12a Assay for Visual and Highly Specific Identification of Zika Virus
Source: J Med Virol. 2026 Apr 15;98(4):e70917. doi: 10.1002/jmv.70917 (PMC13080280; doi:10.1002/jmv.70917)
Supplement: Supplementary file 8 — Supporting File 8 [file JMV-98-e70917-s004.docx]

**Supplementary figure**


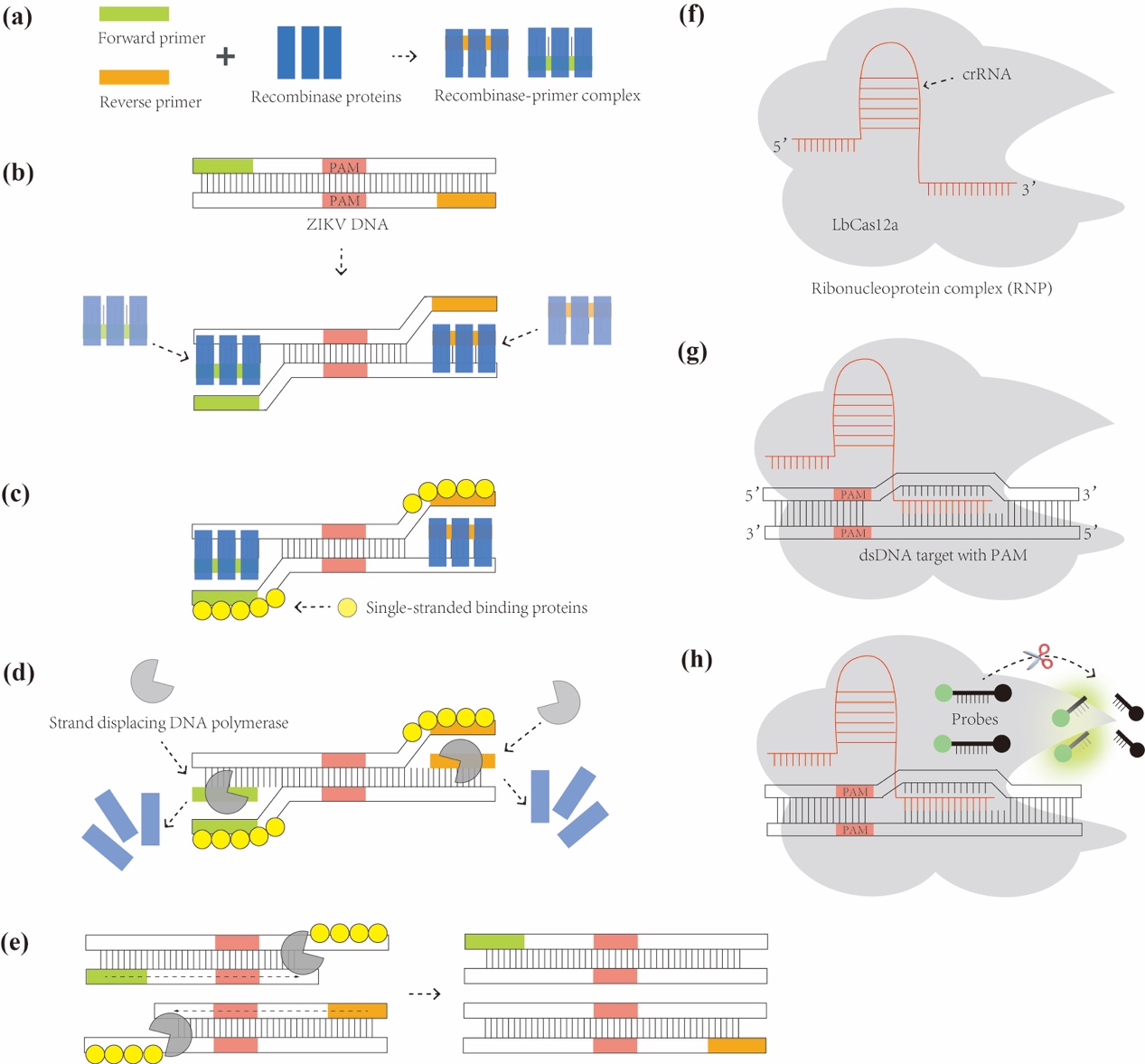


**Supplementary Figure S1. Schematic illustration of the RPA-CRISPR/Cas12a detection mechanism.** (a) Primers form complexes with recombinase; (b) Homologous sequences are located and inserted into the template; (c) Single-stranded binding proteins (SSBs) stabilize the displaced strand; (d) Strand-displacing DNA polymerase initiates synthesis; (e) Amplification proceeds, producing dsDNA; (f) Cas12a binds crRNA to form the ribonucleoprotein (RNP) complex; (g) The RNP binds target DNA at the PAM site; (h) Upon target recognition, Cas12a performs cis-cleavage and activates trans-cleacage of ssDNA reporters (e.g., FAM/BHQ1-labeled), generating fluorescence.


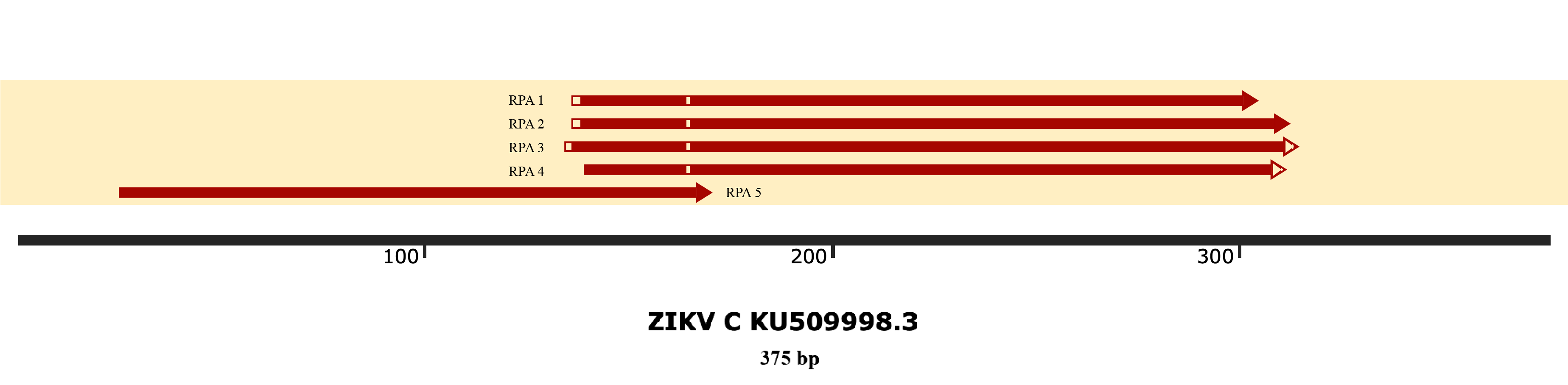


**Supplementary Figure S2. Sequencing-based validation of RPA amplicon specificity.** The RPA amplification products generated by different primer sets were subjected to Sanger sequencing. The obtained sequences were aligned with the reference ZIKV template sequence (GenBank accession no. KU509998.3) used for primer design.


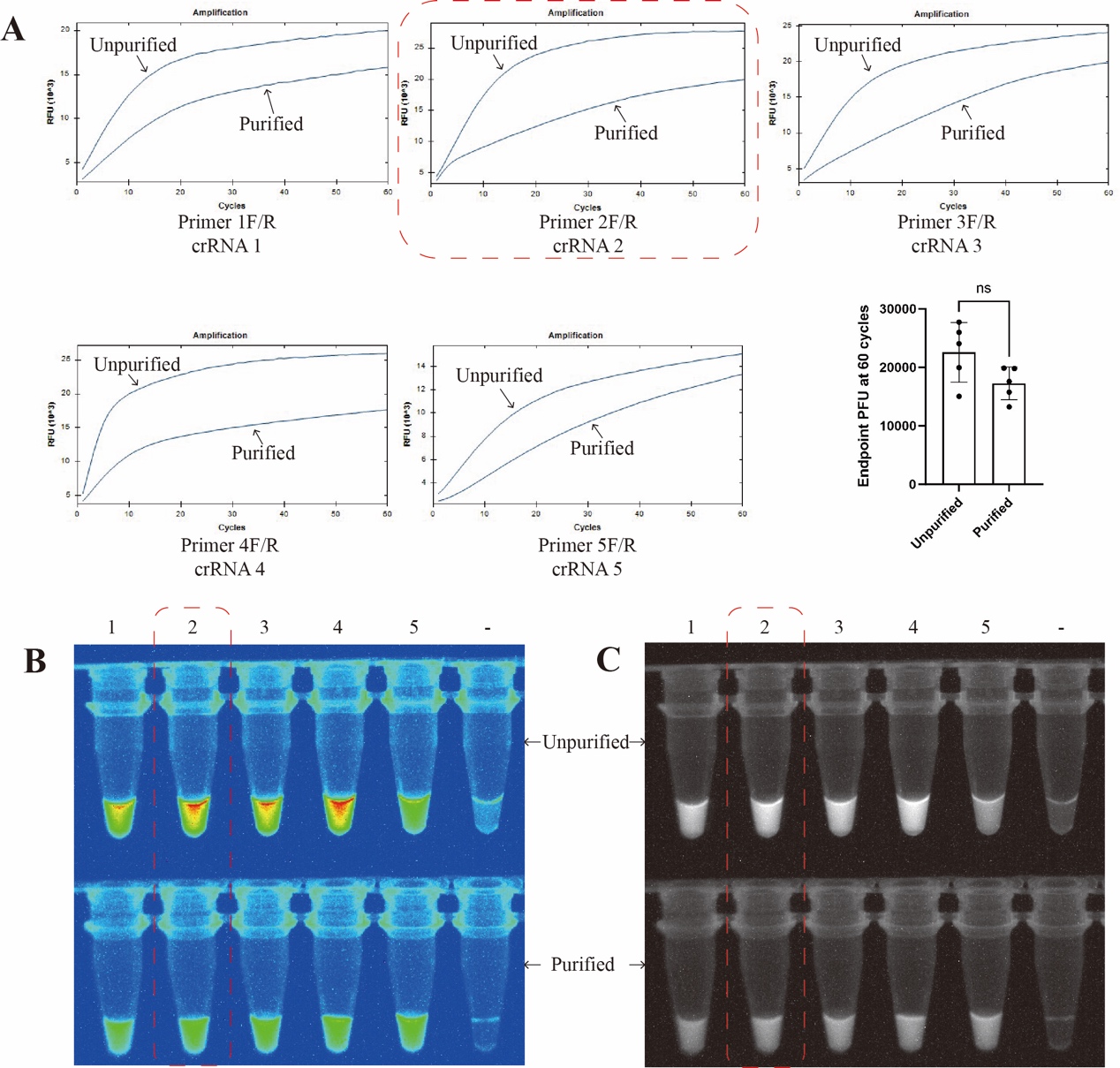


**Supplementary Figure S3.** **Detection results of RPA-CRISPR/Cas12a using purified and unpurified RPA products generated from five primer-crRNA pairs.** (A) Real-time fluorescence detection results of CRISPR/Cas12a using purified and unpurified RPA products (ns, not significant). (B-C) Fluorescence visualization under UV light of CRISPR/Cas12a detection using purified and unpurified RPA products. Lane 1, primer pair 1F/R with crRNA 1; lane 2, primer pair 2F/R with crRNA 2; lane 3, primer pair 3F/R with crRNA 3; lane 4, primer pair 4F/R with crRNA; lane 5, primer pair 5F/R with crRNA 5; Lane -, negative control.


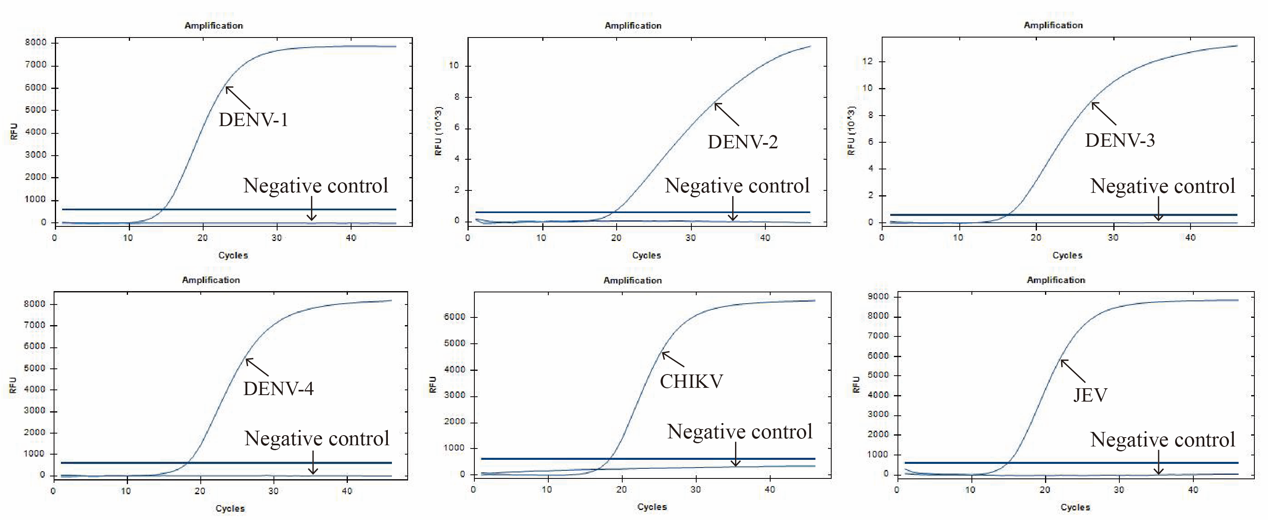


**Supplementary Figure S4. RT-qPCR specificity validation against other arboviruses.** The specificity of the assay was further evaluated using RT-qPCR with RNA templates from six additional arboviruses, including DENV-1, DENV-2, DENV-3, DENV-4, CHIKV, and JEV. As shown by the amplification curves, clear positive amplification signals were observed only for the corresponding viral targets, whereas no amplification was detected in the negative controls.


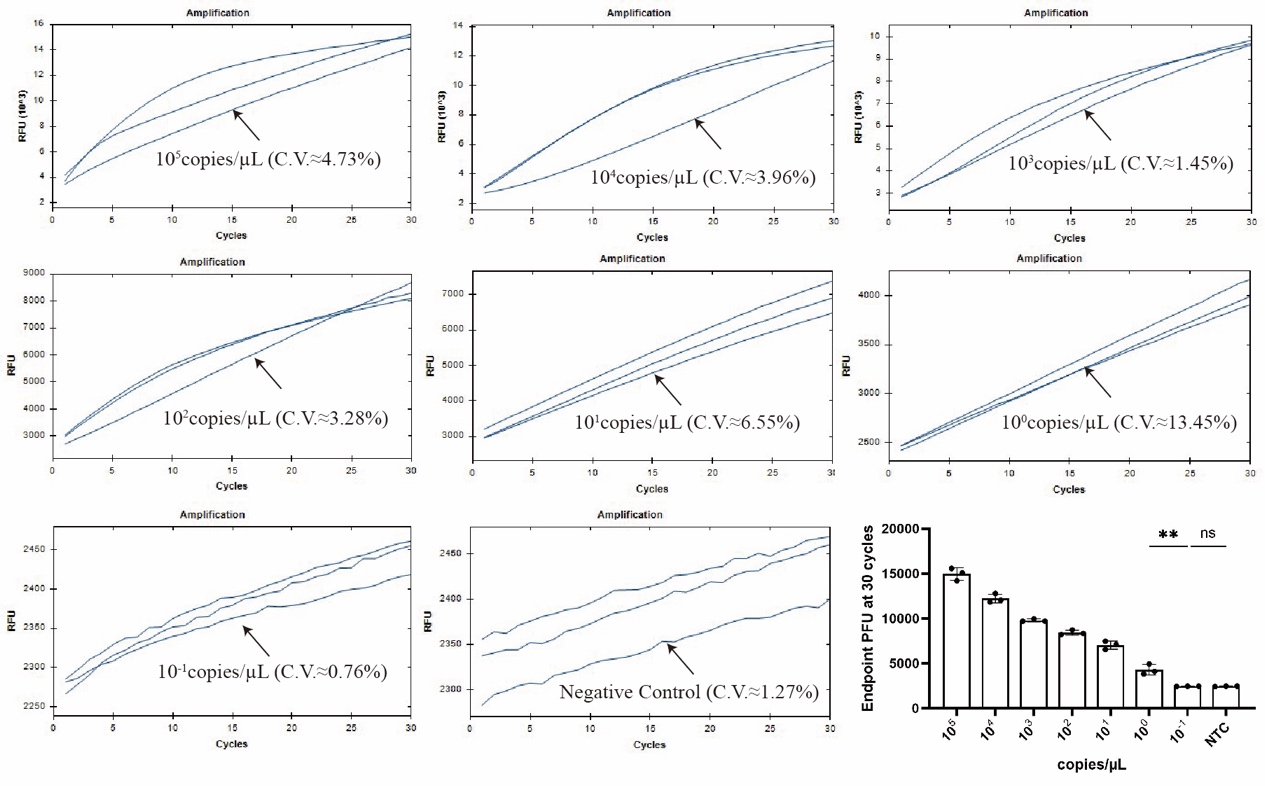


**Supplementary Figure S5.** **Reproducibility evaluation of the RPA–CRISPR/Cas12a detection system.** Real-time fluorescence amplification curves obtained from triplicate reactions at serial target concentrations ranging from 10⁵ to 10^-1^ copies/µL, along with a no-template control (NTC).

And endpoint fluorescence intensities corresponding to each concentration, presented as mean ± SD from three independent replicates. Statistical significance was assessed where indicated (**p < 0.01; ns, not significant).


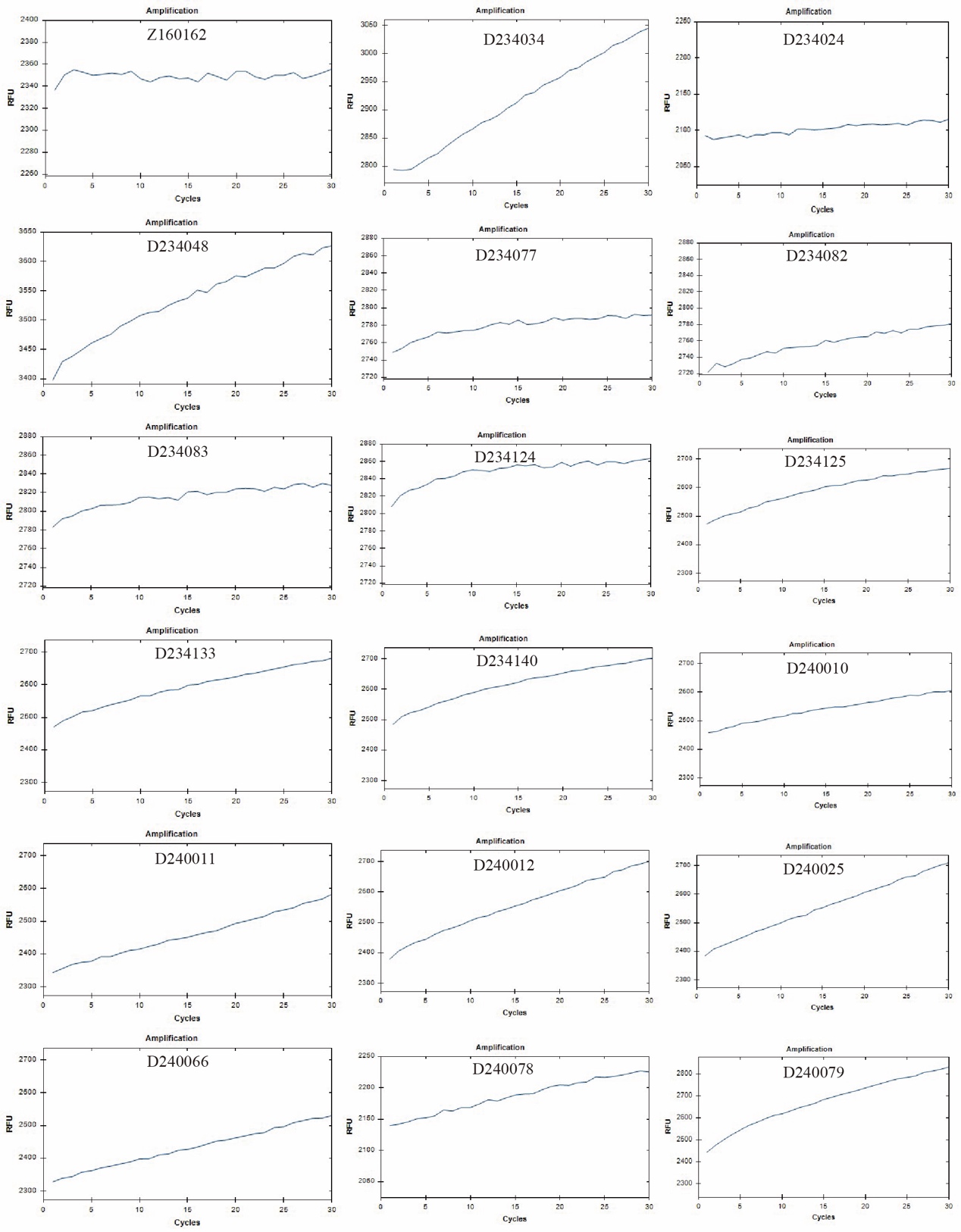

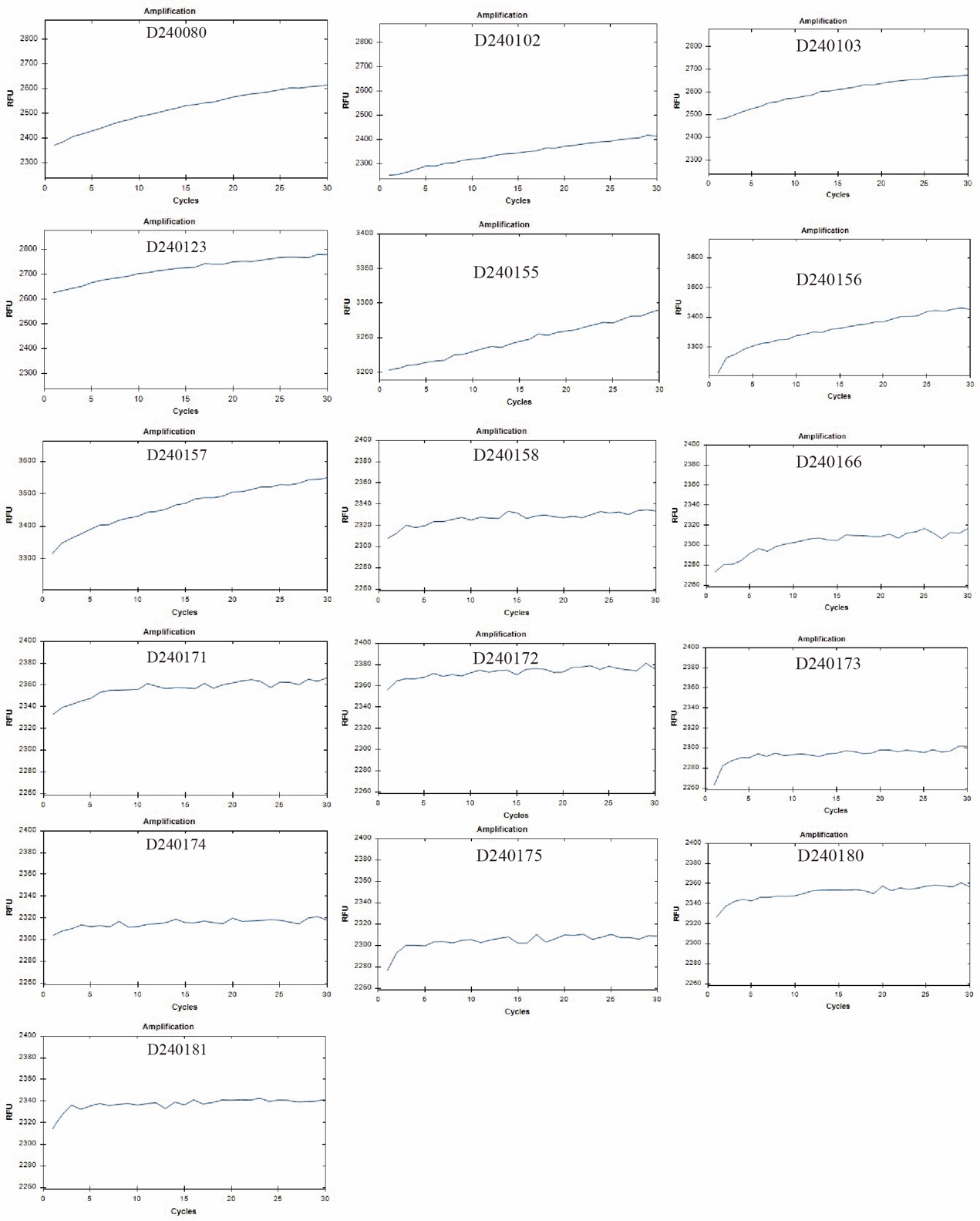


**Supplementary Figure S6. RPA–CRISPR/Cas12a detection results for ZIKV-negative clinical serum samples.** Real-time fluorescence amplification curves of 34 ZIKV-negative clinical serum samples analyzed using the RPA–CRISPR/Cas12a assay. All samples exhibited low and stable fluorescence signals throughout the reaction cycles, with no detectable amplification, consistent with the qRT-PCR results and confirming the absence of ZIKV infection.
